# Beetroot mineral composition affected by mineral and organic fertilization

**DOI:** 10.1371/journal.pone.0221767

**Published:** 2019-09-06

**Authors:** Marko Petek, Nina Toth, Marija Pecina, Tomislav Karažija, Boris Lazarević, Igor Palčić, Szilvia Veres, Mirjana Herak Ćustić

**Affiliations:** 1 Department of Plant Nutrition, Faculty of Agriculture, University of Zagreb, Zagreb, Croatia; 2 Department of Vegetable Crops, Faculty of Agriculture, University of Zagreb, Zagreb, Croatia; 3 Department of Plant Breeding, Genetics, and Biometrics, Faculty of Agriculture, University of Zagreb, Zagreb, Croatia; 4 Centre of Excellence for Biodiversity and Molecular Plant Breeding, Zagreb, Croatia; 5 Institute of Agriculture and Tourism, Poreč, Croatia; 6 Department of Agricultural Botany, Plant Physiology and Biotechnology, Institute of Crop Sciences, University of Debrecen, Debrecen, Hungary; Universidad Nacional Autonoma de Mexico Instituto de Investigaciones en Ecosistemas y Sustentabilidad, MEXICO

## Abstract

In modern agriculture, besides providing high and stable yields, it is imperative to produce products with a high nutritive quality. The goal of this study was to determine the effect of different fertilization regimes on the macro- and micronutrients in beetroot. A 3-year field trial was set up according to a Latin square method with four types of fertilization (unfertilized control, 50 t stable manure ha^−1^, and 500 and 1,000 kg NPK 5-20-30 ha^−1^). The mineral content was determined as follows (mg 100 g^−1^ in fresh weight of beetroot): 14–29 P, 189–354 K, 18–34 Ca, 17–44 Mg, 0.67–1.83 Fe, 0.41–0.65 Mn and 0.28–0.44 Zn. The highest beetroot P content was determined for the treatment with stable manure, especially in a year with dry climatic conditions. The highest beetroot K content was determined for the treatment with 1,000 kg NPK 5-20-30 ha^−1^, but at the same time for the same treatment, a general decreasing trend of micronutrient content was determined, due to the possible antagonistic effect of added potassium. For better mineral status of beetroot, application of combined mineral and organic fertilizers supplemented with additional foliar application of micronutrients can be suggested.

## Introduction

Today, the challenge is no longer the production of agricultural products that are high-yielding only, but also those that have high quality, especially nutritional. Efficient vegetable production is based on large investments, including in fertilizers, and optimizing plant nutrition is essential in achieving high yields and product quality. Imbalances of all nutrients can increase the risk of environmental damage [1] as well as reduce plant growth. Only a well-nourished plant can provide enough minerals for human nutrition, which can be achieved by optimal fertilization following the rules of good agricultural practices.

The adoption of suitable fertilizer management strategies often results in large economic benefits to producers [2] and choosing the appropriate strategy is not an easy process due to different soil type and pH [3,4]. Agriculture, as an economic sector, has to carry out its activity with a profit with respect to the basic principles of sustainable agricultural production, with minimal impact on the environment [5] and rational use of fertilizers, pesticides and irrigation systems. The content and quantity of minerals is affected by plant cultivar [6,7], soil conditions [8], weather conditions during the growing season, fertilizer use [9] and harvest maturity state [10], and can be changed by fertilizer application to balance the content of microelements with macroelements [11,12]. In vegetable production, the yield, as well as the commercial and nutritional quality, is affected by NPK fertilizer application [13].

In plants, as well as humans, minerals play an important role in metabolism and affect health. Humans require carbohydrates, lipids and proteins (amino acids), as well as 13 vitamins and 17 minerals. The nutritive value of vegetables is not based on energy or protein content [14,15] only but also on the content of minerals [16,17] which are necessary for maintaining a healthy and well-nourished body.

Due to its nutritional composition, beetroot (red beet) consumption contributes to the improvement and maintenance of human health and helps in the prevention and treatment of various malignancies, leukaemia and the consequences of radiation exposure as well as the regulation of blood pressure, cholesterol and triglycerides. As beetroot contains high levels of iron, it can be useful during pregnancy and menstruation [18,19].

According to all above, the aim of this investigation was to determine the impact of organic and mineral fertilization on the nutrient content of beetroot.

## Materials and methods

### Field work

A field fertilization trial with beetroot (*Beta vulgaris* var. *conditiva* Alef.), cultivar ‘Bikor’, was carried out in Brašljevica (E N: 411107.5 5060488.5; 45°40’42.99” N 15°21’32.17” E) and Hrvatsko Polje (E N: 395207.625 4973293.5; 44°53’30.64” N 15°10’23.90” E) (Croatia) from 2003 to 2005 (Brašljevica in 2003, B-2003; Hrvatsko Polje in 2004, HP-2004, and in 2005, HP-2005) using the Latin square method with four treatments (unfertilized control, 50 t stable manure ha^−1^, 500 and 1,000 kg NPK 5-20-30 ha^−1^). Untreated beetroot seed was sown (22^nd^ May 2003, 21^st^ May 2004 and 29^th^ June 2005) directly into soil with a plant spacing of 0.07 m × 0.40 m and a main plot area of 12 m^2^. Beetroot were harvested after ~90 days (21^st^ Aug 2003, 24^th^ Aug 2004 and 28^th^ Sep 2005).

The field study was carried out on private land and owners of the land on both locations gave permission to conduct the study on their land properties. During the field study none of endangered or protected species were involved. No specific permissions were required for conducting the field study because it was not carried out in protected area.

### Chemical plant analysis

The edible parts of six plants from each plot at harvest were randomly selected for analysis. Samples of plant material (dried at 105°C) were analysed in triplicate and the results presented as mean values. Prior to digestion the samples were re-dried in order to remove possible moisture gained before the analyses. After digestion of plant material with concentrated HNO_3_ (MILESTONE 1200 Mega Microwave Digester), the phosphorus content was determined using a spectrophotometer, and potassium using a flame photometer, while calcium, magnesium, iron, zinc and manganese were analysed using an atomic absorption spectrophotometer (AAS) [20].

### Chemical soil analysis

Air-dried, ground and homogenized soil was analysed according to the following methods: soil pH was determined electrometrically using a combined electrode (pH-meter MA5730) for a soil : water suspension (1 : 2.5, w/v) (active acidity) [21]; humus by the Tjurin method [22]; potassium and phosphorus by the Egner–Riehm–Domingo method [23]; and nitrogen by the Kjeldahl method [20].

### Climate conditions

Climate data consist of total daily precipitation (mm) and mean daily temperature (°C) and were collected and delivered by the Croatian Meteorological and Hydrological Service for the closest meteorological stations: Jastrebarsko for Brašljevica, and Otočac for Hrvatsko Polje. Multiannual (1961–1991) climate data are organized as total monthly precipitation (mm) and average monthly temperature (°C). Climate data for the years investigated are organized as total decade precipitation (mm) and mean decade temperature (°C), represented in the form of Walter climate diagrams.

### Statistical data analysis

Statistical data analyses were performed using the SAS 8.2 System (2002–2003). Analysis of variance (ANOVA) was performed following a Latin square experimental design. A Tukey’s multiple comparison test (Tukey’s HSD) was applied applied to identify differences among treatments (e.g., fertilization, environment, and their interactions). Normality of the collected data was tested by checking the residual plots and homogeneity of variance (homoscedasticity) by plotting of residuals against fitted values.

## Results

### Chemical soil analysis

Field investigations were carried out on silty loam soil with a soil reaction (pH_H2O_) of 6.1–6.6, with low to moderate humus and nitrogen content, poor in phosphorus and low to rich in potassium (Table 1).

**Table 1:** Chemical properties of the soils collected.

Environment^x^	pH_H2O_	%		AL–mg 100 g^−1^	
		humus	N	P_2_O_5_	K_2_O
B-2003	6.5	2.17	0.12	0.1	6.0
HP-2004	6.1	2.65	0.13	1.5	15.3
HP-2005	6.6	3.10	0.16	6.2	32.8

^x^B-2003 –Brašljevica, 2003; HP-2004 –Hrvatsko Polje, 2004; HP-2005 –Hrvatsko Polje, 2005

### Climate conditions

The closest meteorological station to Brašljevica is Jastrebarsko and to Hrvatsko Polje is Otočac.

**Fig 1:**
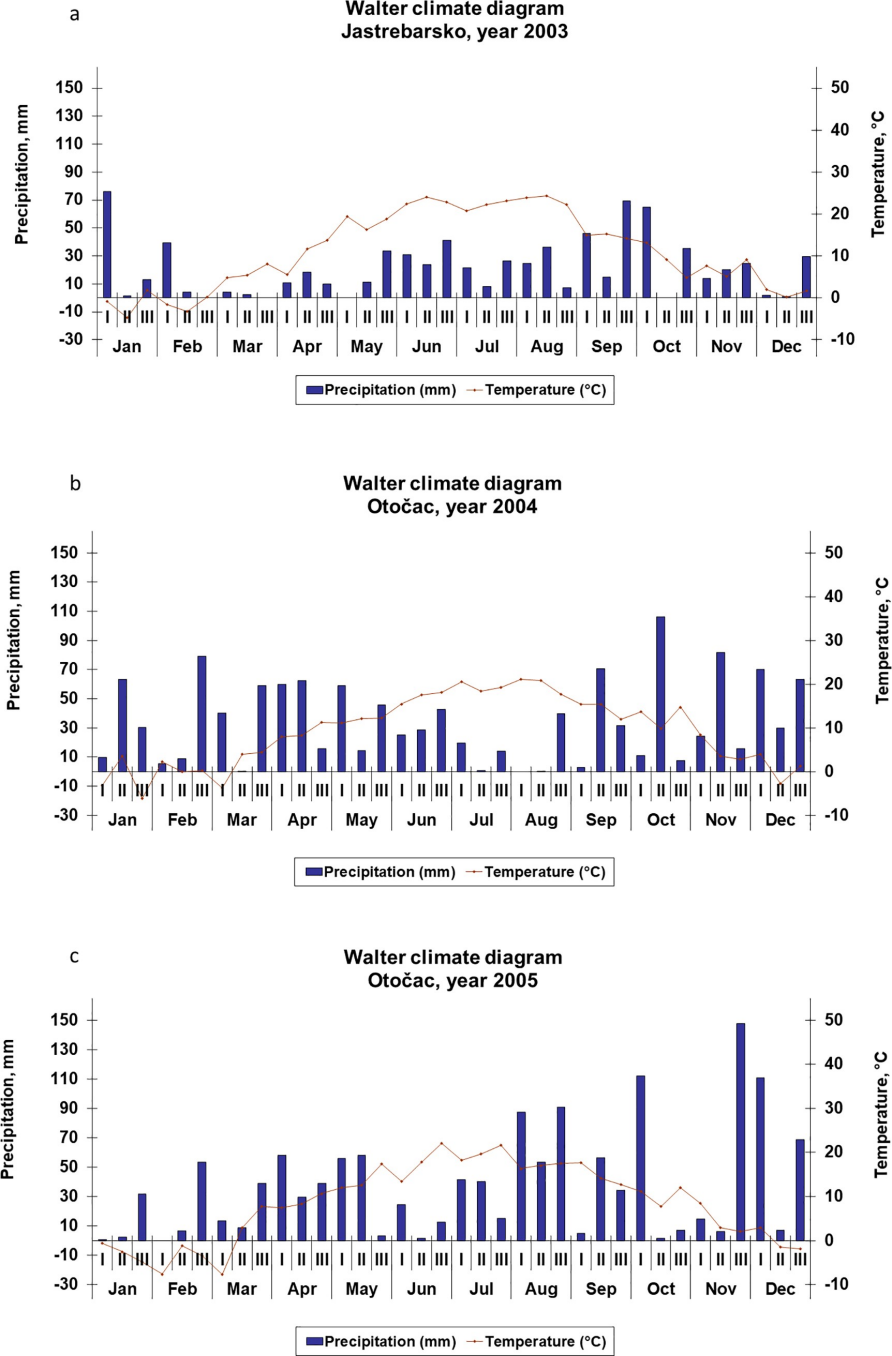
(a, b and c). Walter Climate Diagrams for Jastrebarsko (Brašljevica Location) and Otočac (Hrvatsko Polje Location) Meteorological Stations.

The total precipitation throughout 2003 (Fig 1A) was 766 mm, which is less than the multiannual average (935 mm; Table 2). Precipitation during the vegetation months of red beet growth was 247 mm. In the third decade of May, precipitation was 34 mm, in June 96 mm, in July 56 mm and in the first two decades of August 61 mm. Mean daily air temperature during the period of beetroot growth was 19–23°C. There was an arid period from the beginning of February, so plants were not able to use the water reserves from the soil.

**Table 2:** Multiannual (1961–1991) climate data for Jastrebarsko (Brašljevica Location) and Otočac (Hrvatsko Polje Location) meteorological stations.

Months	Jan	Feb	Mar	Apr	May	Jun	Jul	Aug	Sep	Oct	Nov	Dec
	Jastrebarsko											
TMP*	54	51	60	70	74	100	78	87	105	92	88	76
AMT**	−0.4	1.1	5.9	10.6	15.6	18.7	20.7	20.2	15.6	10.8	4.9	0.9
	Otočac											
TMP*	79	68	75	89	86	77	47	81	127	113	137	127
AMT**	−1.0	0.1	3.9	8.9	14.1	17.8	19.7	19.0	13.9	10.5	5.0	0.1

*TMP–total monthly precipitation (mm)

**AMT–mean monthly temperature (°C)

In 2004 (Fig 1B), weather conditions during the growing season were favourable for beetroot growing thanks to the reserves of soil water before the growing period as well as to the rain during the first half of the growing period. Total precipitation during the year was 1238 mm, which is 133 mm more than the multiannual average (1105 mm, Table 2). During the growing period, precipitation was unevenly distributed. In the last decade of May, precipitation was 46 mm, in June 96 mm, in July 34 mm and in the first two decades of August 0.4 mm. Air temperature during the growing period gradually increased, from 12°C in May to 21°C in August. Generally, temperatures were lower than in 2003, and the ratio between temperature and precipitation was good and had a favourable effect on the growth and development of beetroot.

The total precipitation in 2005 was 1339 mm, 234 mm more than the multiannual average (1105 mm, Table 2). Also, during the vegetation period (July–September), the total precipitation was 423 mm (a lot at the end of vegetation period: in August 231 mm and in September 95 mm) (Fig 1C). Temperatures were favourable for the growth of beetroot. From the beginning to the end of the vegetation period, daily mean temperature decreased from 20°C in July and 17°C in August to 15°C in September. During the whole growing period, the precipitation/temperature ratio was favourable, except in August, when precipitation was significantly higher than required.

### Beetroot macro- and microelement content

In the present study the effect of different fertilization in different environments was evaluated. Table 3 shows the content of dry weight (DW) and content of macroelements (P, K, Ca and Mg) of beetroot in fresh weight, while Table 4 shows the content of microelements (Fe, Mn and Zn) of beetroot in fresh weight according to different fertilization treatments. The results of analysis of variance (ANOVA) of fertilization treatments according to environments are shown in Table 5, while in Table 6 results of combined analysis of variance of experiment in three environments are shown.

**Table 3:** Beetroot dry weight (%) and macroelement (P, K, Ca and Mg) content (mg 100 g^−1^ fresh weight) regarding fertilization treatments and environments.

Fertilization treatments	% Dry Weight			
	B-2003^x^	HP-2004	HP-2005	Average
Control	7.1	14.7	7.3	**9.7**
50 t stable manure ha^−1^	6.2	13.9	6.3	**8.8**
500 kg NPK ha^−1^	6.7	15.0	7.0	**9.6**
1,000 kg NPK ha^−1^	6.9	15.6	7.3	**9.9**
Average	**6.7 B**^**y**^	**14.8 A**	**7.0 A**	
	mg P 100 g^−1^ in fresh weight			
Control	14	26	27	**22**
50 t stable manure ha^−1^	17	30	25	**24**
500 kg NPK ha^−1^	13	29	24	**22**
1,000 kg NPK ha^−1^	13	30	27	**23**
Average	**14 B**	**29 A**	**26 A**	
	mg K 100 g^−1^ in fresh weight			
Control	202	330	324	**285**
50 t stable manure ha^−1^	179	357	301	**279**
500 kg NPK ha^−1^	183	359	319	**287**
1,000 kg NPK ha^−1^	191	368	340	**300**
Average	**189 C**^**y**^	**354 A**	**321 B**	
	mg Ca 100 g^−1^ in fresh weight			
Control	29	36	20	**28**
50 t stable manure ha^−1^	30	28	16	**25**
500 kg NPK ha^−1^	27	27	18	**24**
1,000 kg NPK ha^−1^	28	33	19	**27**
Average	**29 A**^**y**^	**31 A**	**18 B**	
	mg Mg 100 g^−1^ in fresh weight			
control	49	34	17	**33**
50 t stable manure ha^−1^	44	30	16	**30**
500 kg NPK ha^−1^	42	32	17	**30**
1,000 kg NPK ha^−1^	41	31	18	**30**
Average	**44 A**^**y**^	**32 B**	**17 C**	

^x^B-2003 –Brašljevica, 2003; HP-2004 –Hrvatsko Polje, 2004; HP-2005 –Hrvatsko Polje, 2005

^y^Factor level means accompanied by different letters are significantly different, with error p ≤ 0.05 according to Tukey’s HSD test. Small-case letters refer to fertilization treatments. Capital letters refer to average values for environments.

**Table 4:** Beetroot microelement (Fe, Mn and Zn) content (mg 100 g^−1^ fresh weight) regarding fertilization treatments and environments.

Fertilization treatments	mg Fe 100 g^−1^ in fresh weight			
	B-2003^x^	HP-2004	HP-2005	Average
Control	2.23	1.60	0.71	**1.51**
50 t stable manure ha^−1^	1.66	1.41	0.61	**1.23**
500 kg NPK ha^−1^	1.85	1.46	0.65	**1.32**
1,000 kg NPK ha^−1^	1.56	1.53	0.70	**1.26**
Average	**1.83 A**^**y**^	**1.50 B**	**0.67 C**	
	mg Mn 100 g^−1^ in fresh weight			
Control	0.76 a^y^	0.68	0.45	**0.63**
50 t stable manure ha^−1^	0.51 b	0.59	0.36	**0.49**
500 kg NPK ha^−1^	0.67 ab	0.67	0.40	**0.58**
1,000 kg NPK ha^−1^	0.65 ab	0.65	0.44	**0.58**
Average	**0.65 A**	**0.65 A**	**0.41 B**	
	mg Zn 100 g^−1^ in fresh weight			
Control	0.42 a^y^	0.44	0.30	**0.39**
50 t stable manure ha^−1^	0.31 b	0.40	0.25	**0.32**
500 kg NPK ha^−1^	0.37 ab	0.47	0.29	**0.38**
1,000 kg NPK ha^−1^	0.33 ab	0.44	0.28	**0.35**
Average	**0.36 B**	**0.44 A**	**0.28 C**	

^x^B-2003 –Brašljevica, 2003; HP-2004 –Hrvatsko Polje, 2004; HP-2005 –Hrvatsko Polje, 2005

^y^Factor level means accompanied by different letters are significantly different, with error p ≤ 0.05 according to Tukey’s HSD test. Small-case letters refer to fertilization treatments. Capital letters refer to average values of environments.

**Table 5:** Results of analysis of variance (ANOVA) of fertilization treatments according to environments for investigated beetroot properties.

Beetroot properties		Environments		
		B-2003^x^	HP-2004	HP-2005
Dry Weight	d.f.	3	3	3
	Sum of Squares	2.16	5.78	2.68
	Mean of Squares	0.72	1.93	0.89
	F_exp_	2.58	1.65	1.04
	*p* value	0.1495	0.2749	0.4416
mg P 100 g^−1^ in Fresh Weight	d.f.	3	3	3
	Sum of Squares	33.69	53.25	23.69
	Mean of Squares	11.23	17.75	7.9
	F_exp_	1.61	3.00	0.30
	*p* value	0.2835	0.1170	0.824
mg K 100 g^−1^ in Fresh Weight	d.f.	3	3	3
	Sum of Squares	1230.69	3209.19	3124.69
	Mean of Squares	410.23	1069.73	1041.56
	F_exp_	0.63	1.44	0.96
	*p* value	0.6233	0.3216	0.4686
mg Ca 100 g^−1^ in Fresh Weight	d.f.	3	3	3
	Sum of Squares	24.5	239.19	38.69
	Mean of Squares	8.17	79.73	12.9
	F_exp_	1.53	2.38	1.30
	*p* value	0.3001	0.1690	0.3568
mg Mg 100 g^−1^ in Fresh Weight	d.f.	3	3	3
	Sum of Squares	126.50	39.50	9.50
	Mean of Squares	42.17	13.17	3.17
	F_exp_	2.16	1.37	1.31
	*p* value	0.1936	0.3379	0.3548
mg Fe 100 g^−1^ in Fresh Weight	d.f.	3	3	3
	Sum of Squares	1.05	0.09	0.03
	Mean of Squares	0.35	0.03	0.01
	F_exp_	1.62	1.62	0.97
	*p* value	0.2808	0.2811	0.4655
mg Mn 100 g^−1^ in Fresh Weight	d.f.	3	3	3
	Sum of Squares	0.13	0.02	0.02
	Mean of Squares	0.04	0.01	0.01
	F_exp_	7.57	1.71	2.92
	*p* value	0.0183	0.2628	0.1225
mg Zn 100 g^−1^ in Fresh Weight	d.f.	3	3	3
	Sum of Squares	0.03	0.1	0.01
	Mean of Squares	0.010	0.004	0.001
	F_exp_	5.82	3.6	0.92
	*p* value	0.0329	0.0851	0.4858

^x^B-2003 –Brašljevica, 2003; HP-2004 –Hrvatsko Polje, 2004; HP-2005 –Hrvatsko Polje, 2005

**Table 6:** Results of combined analysis of variance of experiment in three environments for investigated beetroot properties.

Beetroot properties		Fertilization	Environment	Fertilization x Environment
Dry Weight	d.f.	3	2	6
	Sum of Squares	9.19	672.49	1.42
	Mean of Squares	3.06	336.25	0.24
	F_exp_	12.93	213.74	0.31
	*p* value	0.0050	0.0006	0.9244
mg P 100 g^−1^ in Fresh Weight	d.f.	3	2	6
	Sum of Squares	24.17	1864.54	86.46
	Mean of Squares	8.06	932.27	14.41
	F_exp_	0.56	5.09	1.10
	*p* value	0.6613	0.0335	0.3975
mg K 100 g^−1^ in Fresh Weight	d.f.	3	2	6
	Sum of Squares	2704.56	243807	4860.00
	Mean of Squares	901.52	121904	810.00
	F_exp_	1.11	43.44	0.98
	*p* value	0.4147	0.0001	0.4666
mg Ca 100 g^−1^ in Fresh Weight	d.f.	3	2	6
	Sum of Squares	164.17	1538.29	138.21
	Mean of Squares	54.72	769.15	23.03
	F_exp_	2.38	17.18	1.42
	*p* value	0.169	0.0027	0.2622
mg Mg 100 g^−1^ in Fresh Weight	d.f.	3	2	6
	Sum of Squares	100.17	5960.67	75.33
	Mean of Squares	33.39	2980.33	12.56
	F_exp_	2.66	52.44	1.2
	*p* value	0.1422	<0.0001	0.3526
mg Fe 100 g^−1^ in Fresh Weight	d.f.	3	2	6
	Sum of Squares	0.59	11.49	0.57
	Mean of Squares	0.2	5.75	0.10
	F_exp_	2.07	23.44	1.18
	*p* value	0.2059	0.0032	0.3611
mg Mn 100 g^−1^ in Fresh Weight	d.f.	3	2	6
	Sum of Squares	0.13	0.58	0.04
	Mean of Squares	0.004	0.29	0.01
	F_exp_	7.33	9.10	1.53
	*p* value	0.0198	0.0056	0.2239
mg Zn 100 g^−1^ in Fresh Weight	d.f.	3	2	6
	Sum of Squares	0.03	0.20	0.01
	Mean of Squares	0.010	0.102	0.002
	F_exp_	4.86	14.98	1.55
	*p* value	0.0480	0.0011	0.2201

According to results, beetroot obtained the highest (*p* = 0.0006) dry weight content at Hrvatsko Polje in 2004 (14.8% DW) when the climate conditions were favourable, compared to other two environments. Results showed that at Brašljevica in 2003 and Hrvatsko Polje in 2004, the highest average beetroot phosphorus content (24 mg P 100 g^−1^ fresh weight) was determined for the treatment with stable manure compared to other fertilization treatments. Also, the environment had a significant impact on the beetroot phosphorus content, which was higher (*p* = 0.0335) at Hrvatsko Polje in 2004 and 2005 (average 29 and 26 mg P 100 g^−1^ fresh weight, respectively). The highest average beetroot potassium content (300 mg K 100 g^−1^ fresh weight) was obtained for fertilization with 1,000 kg NPK ha^−1^. The same trend was noticed at Hrvatsko Polje in 2004 and 2005. Regardless of fertilization the highest potassium content (*p* = 0.0001) was found at Hrvatsko Polje in 2004 (354 mg K 100 g^−1^ fresh weight). No kind of fertilization in this study showed a positive effect on calcium and magnesium uptake by beetroot, so the highest average content was determined for the control treatment (28 mg Ca 100 g^−1^ fresh weight and 33 mg Mg 100 g^−1^ fresh weight). On the other hand, the environment showed a significant effect on uptake of calcium and magnesium. The highest annual average calcium content (31 mg Ca 100 g^−1^ fresh weight) was found for Hrvatsko Polje in 2004 (*p* = 0.0027) although it was not significantly different to that at Brašljevica in 2003 (29 mg Ca 100 g^−1^ fresh weight). The highest magnesium content (44 mg Mg 100 g^−1^ fresh weight) was found for Brašljevica in 2003 (*p*<0.0001).

The results show that, similar to Ca and Mg content, the highest content of microelements was determined for the control treatment (1.51 mg Fe 100 g^−1^ fresh weight, 0.63 mg Mn 100 g^−1^ fresh weight and 0.39 mg Zn 100 g^−1^ fresh weight), and fertilization had no significant effect. The highest microelement content (Fe, Mn and Zn) was found either at Brašljevica in 2003 and/or at Hrvatsko Polje in 2004 (*p* = 0.0032, *p* = 0.0056 and *p* = 0.0011, respectively).

## Discussion

Beetroot is an important vegetable in the human diet, not just because of its mineral content, but also because of its bioactive compounds such as amino acids, flavonoids and phenols, as well as carotenoids and betalains which, among others, have great anti-oxidative activity [6,24,25]. Minerals affect the synthesis of secondary metabolites directly (as part of their structure) or indirectly (as parts or co-factors or activators of enzymes). Therefore, it is very important to optimize nutrient supply [26] for all plants, which can be done by choosing appropriate and sustainable fertilization design.

Many authors have reported the positive effect of fertilization with mineral and/or organic fertilizers on the nutritive value and yield of vegetables: cabbage and spinach [27,28], eggplant [29], common bean [30], carrot [31] and red head chicory [32].

In the literature, different recommendations for beetroot fertilization can be found: 150 kg N ha^−1^, 50 kg P ha^−1^, 220 kg K ha^−1^ and 40 kg Mg ha^−1^ [19]; 60 kg N ha^−1^, 80 kg P_2_O_5_ ha^−1^ and 150 kg K_2_O ha^−1^ [16]; or 85–110 kg N ha^−1^, 50–170 kg P_2_O_5_ ha^−1^ and 50–170 kg K_2_O ha^-1^ [33]; in this present research as mineral fertilization treatments we used 25 kg N ha^−1^, 100 kg P_2_O_5_ ha^−1^ and 150 kg K_2_O ha^−1^ (as 500 kg NPK 5-20-30 ha^−1^) and 50 kg N ha^−1^, 200 kg P_2_O_5_ ha^−1^ and 300 kg K_2_O ha^−1^ (as 1,000 kg NPK 5-20-30 ha^-1^). However, it is worth highlighting that any fertilization recommendation for serious agriculture production should be based on both the chemical composition of the soil and plant species nutrient need for the desired yield, taking into account cultivar characteristics.

Statistical analyses showed variations in the quantity of certain minerals with respect to the research environments, which were dependent upon the initial soil nutrient content and subject to weather conditions (total precipitation and mean daily temperature) as well as differences in the nutrient-holding capacity of the soil on which the study was conducted.

The nutrient status of soils and crops can be affected by different fertilization. Results showed that organic fertilization had a positive effect on beetroot P content. Organic fertilization increases the availability of phosphorus in soil [34] as during the mineralization process a certain amount of P is released from organic compounds [35]. The highest beetroot P content was determined for the treatment with stable manure, and was more pronounced in the year with less precipitation (2003). Therefore, increased soil organic matter content has a positive effect in extreme climate conditions due to its favourable effect on soil water-air properties [4,36].

In this study, organic fertilization had no significant effect on microelement uptake, a result that was reported by some other authors too [37,38], although it would have been expected that the application of stable manure would increase the uptake of the microelement through the formation of chelators, and following the chelates, during the mineralization process. On the contrary, some other studies reported significantly increased availability of Zn, Fe and Mn [39] and overall soil fertility [40] by organic fertilization. Also, the DTPA-extractable soil Zn, Fe and Mn concentrations were increased from 0.41 to 1.08 mg kg^−1^, from 10.3 to 17.7 mg kg^−1^, and from 9.7 to 11.8 mg kg^−1^, respectively, with increasing soil organic matter content, thus showing the importance of soil organic matter in micronutrient availability for crops [41]. Finally, despite there being no effect of organic fertilization, the microelement values obtained for beetroot are higher than almost all the literature data shown in Table 7.

**Table 7:** Beetroot macro- and micronutrient status according to different authors.

Authors	mg 100 g^−1^ in fresh weight						
	P	K	Ca	Mg	Fe	Mn	Zn
Lisiewska et al. (2006) [16]					0.38	0.39	0.60
Ekholm et al. (2007) [17]	24	345	13	21	0.38	0.40	0.21
Yashwant (2015) [25]	38	305	16	23	0.79		0.35
Maynard and Hochmuth (1997) [32]					0.90		
Grembecka et al. (2008) [42]					0.99		0.26
Kołota and Adamczewska-Sowinska (2006) [43]	48	324	16		0.90		
Lindow and Peterson (1927) [44]						0.62–1.35	
Siener et al. (2006) [45]					0.90		
This research							
Range	13–30	179–368	16–36	16–49	0.61–2.23	0.36–0.76	0.25–0.47
Average	23	288	26	31	1.33	0.57	0.36

Furthermore, not just the total content of nutrients in the soil, but the interaction of soil macronutrients and micronutrients affect micronutrients uptake [46]. In our study, the soil potassium content had a big role (most probably due to the high added potassium quantity) in decreasing uptake of the macroelements Ca and Mg, as potassium has an antagonistic relationship with those elements [47–49], although there are some findings that claim the opposite [50]. So, in mineral fertilizer treatments, a negative effect on uptake of those cations was observed, due to the tendency of plants to maintain a constant amount of total cations [50] and the amount of potassium ion in the soil being much higher than that of other cations.

Nevertheless, the genetic traits of a beetroot cultivar have a great effect on its mineral composition without any differences in fertilization treatments [6]. As in our study only one cultivar (‘Bikor’) was used, we could assume that some other cultivar may have a better response to fertilization treatments.

Comparing climatological conditions in all three investigation years during the growing period, it is evident that the most favourable conditions for normal growth and development of beetroot prevailed in 2004; 2003 was relatively unfavourable because of low precipitation and a poor ratio of temperature and precipitation, as was 2005 with an increased precipitation rate. The results show that weather conditions have a significant effect on the mineral composition of beetroot, and also on the yield. A previous paper [51] showed that in dry conditions (in 2003), application of 50 t stable manure ha^−1^ increased yield up to three times compared to the control treatment. On the contrary, mineral fertilization showed better results in years with optimal precipitation. However, in rainy 2005, the nutrient uptake determined was at a lower level due to possible leaching of nutrients [52]. All this suggests that the best results could be expected with the combined application of organic and mineral fertilizers [53,54], as well as microelements, especially by foliar fertilizers [55] in climatic extreme conditions.

As previously mentioned, not only plants need an optimal dose of nutrients for normal growth and development, but also humans. Table 8 shows the recommended daily intake of macro- and micronutrients for humans by age and sex. Regardless of the various data obtained, beetroot from this research is an extremely valuable food because it is a very good source of minerals. This thesis supports the fact that daily consumption of 100 g of beetroot (from our current study) can assure 2–4% of the daily requirement of phosphorus, 9–18% of potassium, 2–4% of calcium, 5–12% of magnesium, 5–13% of iron, 21–33% of manganese and 3–4% of zinc according to Regulation (EU) No 1169/2011 [56].

**Table 8:** Recommended daily intake of macro- and micronutrients according to different sources.

Source	Sex/age	mg day^−1^						
		P	K	Ca	Mg	Fe	Mn	Zn
Regulation (EU) No 1169/2011 [56]		700	2000	800	375	14	2	10
Flynn et al. (2003) [57]		800		800	300	14		15
Institute of Medicine (1997, 2001, 2004) [58–60]	Men age 31–50	700	4700	1,000	320–420	8	2.3	11
	Women age 31–50					18	1.8	8
WHO/FAO (2004) [61]	Men age 19–50				260	13.7		7
	Women age 19–50			1,000	220	29.4		4.9

With all that is presented and discussed, one question will always be asked: what amount of any macro- or micronutrient has to be available to plants in the soil or has to be added to the soil, at what time and in what form to achieve high, stable yields of the desired quality without crossing economic, environmental or energy limits? All that must be considered in order to maintain and improve soil fertility without an adverse impact on the environment, plants, animals and/or humans [46].

So, in agricultural production, it is not possible to expect that fertilization will have a strong effect on plant mineral composition in all conditions, because as it can be seen, numerous different factors affect the open-field food factory.

## Conclusions

Data indicated that the macro- and micronutrient content of beetroot can be affected by the environment in which they are grown to a greater extent than by fertilization. The highest phosphorus, potassium and calcium content was found in beetroot grown at Hrvatsko Polje in 2004 under favourable climatic conditions. The highest phosphorus content was achieved by treatment with 50 t stable manure ha^−1^ (especially in dry climatic conditions), and the highest potassium content with 1,000 kg NPK 5-20-30 ha^−1^. The highest content of the microelements calcium and magnesium in beetroot was determined for the unfertilized treatment, probably due to antagonism with potassium. Therefore, beetroot nutrient status would surely be considerably higher if common fertilization with mineral fertilizers were to be both combined with organic fertilizers and supplemented with additional foliar application of micronutrient fertilizers in order to increase the content of all nutrients to produce a high-value food.

## Supporting information

S1 File All data collected for minerals in beetroot. (PDF)
