# Supplementary material for: Beetroot mineral composition affected by mineral and organic fertilization
Source: PLoS One. 2019 Sep 6;14(9):e0221767. doi: 10.1371/journal.pone.0221767 (PMC6730864; doi:10.1371/journal.pone.0221767)
Supplement: S1 File — (PDF) [file pone.0221767.s001.pdf]

MINERALS IN BEETROOT

2003

ID	sample				yield	%	crude	% in dry matter							mg/kg in dry matter				%
	label	var	rep	rep	t/ha	ST	proteins	N	P_2O_5	P	K_2O	K	Ca	Mg	Fe	Zn	Mn	Cu	Brix
	oznaka za SAS	var	rep1	rep2	prinos_03	ST_03	sirprot_03	N_03	P2O5_03	P_03	K2O_03	K_03	Ca_03	Mg_03	Fe_03	Zn_03	Mn_03	Cu_03	Brix_03
1398	1	**1**	**I**	I	1,2	7,5	16,8	2,68	0,38	0,17	2,98	2,47	0,46	0,61	300	48	99	11,6	7,0
1399	2	**2**	**I**	II	10,6	6,2	17,7	2,83	0,48	0,21	2,78	2,31	0,4	0,63	290	43	75	10,6	9,0
1400	3	**3**	**I**	III	6,0	6,5	17,1	2,74	0,43	0,19	2,96	2,46	0,42	0,51	180	41	97	11,0	8,0
1401	4	**4**	**I**	IV	10,1	6,4	19,1	3,06	0,46	0,20	3,62	3,00	0,41	0,56	170	44	110	11,8	10,0
1402	5	**2**	**II**	IV	8,0	5,5	24,2	3,87	0,56	0,24	3,92	3,25	0,52	0,8	200	62	89	12,8	8,0
1403	6	**4**	**II**	III	10,3	7,1	17,5	2,8	0,28	0,12	3,00	2,49	0,3	0,46	230	40	87	11,0	7,0
1404	7	**1**	**II**	II	4,4	5,8	21,5	3,44	0,49	0,21	3,62	3,00	0,36	0,8	269	66	100	12,0	7,0
1405	8	**3**	**II**	I	6,1	6,8	19,6	3,14	0,5	0,22	3,62	3,00	0,42	0,66	300	55	106	11,0	5,0
1406	9	**2**	**III**	I	12,9	6,6	20,1	3,22	0,62	0,27	3,72	3,09	0,53	0,7	310	38	80	12,0	7,0
1407	10	**4**	**III**	II	6,7	7,3	21,8	3,48	0,51	0,22	3,99	3,31	0,38	0,71	300	54	98	12,1	6,0
1408	11	**1**	**III**	III	3,0	7,9	20,3	3,24	0,48	0,21	3,98	3,30	0,36	0,69	370	56	112	11,0	8,0
1409	12	**3**	**III**	IV	2,8	6,5	23,4	3,75	0,46	0,20	3,72	3,09	0,4	0,69	440	67	118	13,2	7,0
1410	13	**1**	**IV**	IV	3,9	7,3	22,6	3,61	0,46	0,20	3,10	2,57	0,45	0,66	300	69	114	13,2	5,0
1411	14	**2**	**IV**	III	8,3	6,3	23,6	3,77	0,81	0,35	3,60	2,99	0,49	0,76	269	57	86	10,9	5,0
1412	15	**3**	**IV**	II	8,6	7,2	19,4	3,1	0,44	0,19	2,86	2,37	0,36	0,61	190	58	80	11,2	8,0
1413	16	**4**	**IV**	I	5,6	7,0	23,1	3,7	0,45	0,20	2,64	2,19	0,53	0,64	190	50	80	12,6	6,0

		yield	%	crude	% in dry matter							mg/kg in dry matter				%
**Average by variants**		t/ha	ST	proteins	N	P_2O_5	P	K_2O	K	Ca	Mg	Fe	Zn	Mn	Cu	Brix
**varijant 1**	unfertilized control	3,1	7,1	20,3	3,2	0,5	0,20	3,4	2,84	0,4	0,7	310	60	106	12,0	6,8
**varijant 2**	stable manure, 50 t/ha	9,9	6,2	21,4	3,4	0,6	0,27	3,5	2,91	0,5	0,7	267	50	83	11,6	7,3
**varijant 3**	500 kg NPK/ha*	5,9	6,7	19,9	3,2	0,5	0,20	3,3	2,73	0,4	0,6	278	55	100	11,6	7,0
**varijant 4**	1000 kg NPK/ha*	8,2	6,9	20,4	3,3	0,4	0,19	3,3	2,75	0,4	0,6	223	47	94	11,9	7,3

formula: broj/(100/ST) formula: broj/(100/ST)

MINERALS IN BEETROOT

2003

ID	sample				yield	%	crude	% in fresh matter							mg/kg in fresh matter				%
	label	var	rep	rep	t/ha	ST	proteins	N	P_2O_5	P	K_2O	K	Ca	Mg	Fe	Zn	Mn	Cu	Brix
	oznaka za SAS	var	rep1	rep2	prinos_03	ST_03	sirprot_03	N_03	P2O5_03	P_03	K2O_03	K_03	Ca_03	Mg_03	Fe_03	Zn_03	Mn_03	Cu_03	Brix_03
1398	1	**1**	**I**	I	1,2	7,5	1,2	0,20	0,03	0,01	0,22	0,18	0,03	0,05	22,35	3,58	7,38	0,86	7,0
1399	2	**2**	**I**	II	10,6	6,2	1,1	0,18	0,03	0,01	0,17	0,14	0,02	0,04	18,07	2,68	4,67	0,66	9,0
1400	3	**3**	**I**	III	6,0	6,5	1,1	0,18	0,03	0,01	0,19	0,16	0,03	0,03	11,75	2,68	6,33	0,72	8,0
1401	4	**4**	**I**	IV	10,1	6,4	1,2	0,19	0,03	0,01	0,23	0,19	0,03	0,04	10,80	2,79	6,99	0,75	10,0
1402	5	**2**	**II**	IV	8,0	5,5	1,3	0,21	0,03	0,01	0,22	0,18	0,03	0,04	11,08	3,43	4,93	0,71	8,0
1403	6	**4**	**II**	III	10,3	7,1	1,2	0,20	0,02	0,01	0,21	0,18	0,02	0,03	16,31	2,84	6,17	0,78	7,0
1404	7	**1**	**II**	II	4,4	5,8	1,2	0,20	0,03	0,01	0,21	0,17	0,02	0,05	15,60	3,83	5,80	0,70	7,0
1405	8	**3**	**II**	I	6,1	6,8	1,3	0,21	0,03	0,01	0,24	0,20	0,03	0,04	20,28	3,72	7,17	0,74	5,0
1406	9	**2**	**III**	I	12,9	6,6	1,3	0,21	0,04	0,02	0,25	0,20	0,03	0,05	20,43	2,50	5,27	0,79	7,0
1407	10	**4**	**III**	II	6,7	7,3	1,6	0,25	0,04	0,02	0,29	0,24	0,03	0,05	21,96	3,95	7,17	0,89	6,0
1408	11	**1**	**III**	III	3,0	7,9	1,6	0,26	0,04	0,02	0,32	0,26	0,03	0,05	29,38	4,45	8,89	0,87	8,0
1409	12	**3**	**III**	IV	2,8	6,5	1,5	0,24	0,03	0,01	0,24	0,20	0,03	0,04	28,38	4,32	7,61	0,85	7,0
1410	13	**1**	**IV**	IV	3,9	7,3	1,6	0,26	0,03	0,01	0,23	0,19	0,03	0,05	21,87	5,03	8,31	0,96	5,0
1411	14	**2**	**IV**	III	8,3	6,3	1,5	0,24	0,05	0,02	0,23	0,19	0,03	0,05	16,97	3,60	5,43	0,69	5,0
1412	15	**3**	**IV**	II	8,6	7,2	1,4	0,22	0,03	0,01	0,20	0,17	0,03	0,04	13,59	4,15	5,72	0,80	8,0
1413	16	**4**	**IV**	I	5,6	7,0	1,6	0,26	0,03	0,01	0,18	0,15	0,04	0,04	13,26	3,49	5,58	0,88	6,0

			yield	%	crude	% in fresh matter							mg/kg in fresh matter				%
**Average by variants**			t/ha	ST	proteins	N	P_2O_5	P	K_2O	K	Ca	Mg	Fe	Zn	Mn	Cu	Brix
**varijant 1**	unfertilized control		3,1	7,1	1,4	0,2	0,03	0,01	0,2	0,2	0,0	0,0	22	4	8	0,8	6,8
**varijant 2**	stable manure, 50 t/ha		9,9	6,2	1,3	0,2	0,04	0,02	0,2	0,2	0,0	0,0	17	3	5	0,7	7,3
**varijant 3**	500 kg NPK/ha*		5,9	6,7	1,3	0,2	0,03	0,01	0,2	0,2	0,0	0,0	18	4	7	0,8	7,0
**varijant 4**	1000 kg NPK/ha*		8,2	6,9	1,4	0,2	0,03	0,01	0,2	0,2	0,0	0,0	16	3	6	0,8	7,3

formula: broj*1000

makroelementi: formula: broj*1000
mikroelementi: formula: broj/10

MINERALS IN BEETROOT

2003

ID	sample				yield	%	crude	mg/100 g in fresh matter							mg/100 g in fresh matter				%
	label	var	rep	rep	t/ha	ST	proteins	N	P_2O_5	P	K_2O	K	Ca	Mg	Fe	Zn	Mn	Cu	Brix
	oznaka za SAS	var	rep1	rep2	prinos_03	ST_03	sirprot_03	N_03	P2O5_03	P_03	K2O_03	K_03	Ca_03	Mg_03	Fe_03	Zn_03	Mn_03	Cu_03	Brix_03
1398	1	**1**	**I**	I	1,2	7,5	1247,9	199,7	28,3	12,4	222,0	184,2	34,3	45,4	2,24	0,36	0,74	0,09	7,0
1399	2	**2**	**I**	II	10,6	6,2	1101,9	176,3	29,9	13,1	173,2	143,7	24,9	39,2	1,81	0,27	0,47	0,07	9,0
1400	3	**3**	**I**	III	6,0	6,5	1118,3	178,9	28,1	12,3	193,3	160,4	27,4	33,3	1,18	0,27	0,63	0,07	8,0
1401	4	**4**	**I**	IV	10,1	6,4	1214,4	194,3	29,2	12,8	229,9	190,8	26,0	35,6	1,08	0,28	0,70	0,07	10,0
1402	5	**2**	**II**	IV	8,0	5,5	1340,0	214,4	31,0	13,5	217,2	180,2	28,8	44,3	1,11	0,34	0,49	0,07	8,0
1403	6	**4**	**II**	III	10,3	7,1	1240,8	198,5	19,9	8,7	212,7	176,5	21,3	32,6	1,63	0,28	0,62	0,08	7,0
1404	7	**1**	**II**	II	4,4	5,8	1247,0	199,5	28,4	12,4	210,0	174,2	20,9	46,4	1,56	0,38	0,58	0,07	7,0
1405	8	**3**	**II**	I	6,1	6,8	1326,7	212,3	33,8	14,8	244,7	203,1	28,4	44,6	2,03	0,37	0,72	0,07	5,0
1406	9	**2**	**III**	I	12,9	6,6	1326,2	212,2	40,9	17,8	245,1	203,4	34,9	46,1	2,04	0,25	0,53	0,08	7,0
1407	10	**4**	**III**	II	6,7	7,3	1592,1	254,7	37,3	16,3	292,1	242,4	27,8	52,0	2,20	0,40	0,72	0,09	6,0
1408	11	**1**	**III**	III	3,0	7,9	1607,9	257,3	38,1	16,6	316,0	262,3	28,6	54,8	2,94	0,44	0,89	0,09	8,0
1409	12	**3**	**III**	IV	2,8	6,5	1511,7	241,9	29,7	13,0	239,9	199,1	25,8	44,5	2,84	0,43	0,76	0,09	7,0
1410	13	**1**	**IV**	IV	3,9	7,3	1644,8	263,2	33,5	14,6	226,0	187,5	32,8	48,1	2,19	0,50	0,83	0,10	5,0
1411	14	**2**	**IV**	III	8,3	6,3	1486,8	237,9	51,1	22,3	227,2	188,5	30,9	48,0	1,70	0,36	0,54	0,07	5,0
1412	15	**3**	**IV**	II	8,6	7,2	1385,3	221,7	31,5	13,7	204,5	169,7	25,7	43,6	1,36	0,41	0,57	0,08	8,0
1413	16	**4**	**IV**	I	5,6	7,0	1614,1	258,3	31,4	13,7	184,3	152,9	37,0	44,7	1,33	0,35	0,56	0,09	6,0

		yield	%	crude	mg/100 g in fresh matter							mg/100 g in fresh matter				%
**Average by variants**		t/ha	ST	proteins	N	P_2O_5	P	K_2O	K	Ca	Mg	Fe	Zn	Mn	Cu	Brix
**varijant 1**	unfertilized control	3,1	7,1	1437	230	32	14	243	202	29	49	2,23	0,42	0,76	0,08	6,8
**varijant 2**	stable manure, 50 t/ha	9,9	6,2	1314	210	38	17	216	179	30	44	1,66	0,31	0,51	0,07	7,3
**varijant 3**	500 kg NPK/ha*	5,9	6,7	1335	214	31	13	221	183	27	42	1,85	0,37	0,67	0,08	7,0
**varijant 4**	1000 kg NPK/ha*	8,2	6,9	1415	226	29	13	230	191	28	41	1,56	0,33	0,65	0,08	7,3

MINERALS IN BEETROOT

2004 svježa cikla u berbi

ID	sample				yield	%	crude	% in dry matter							mg/kg in dry matter				%
	label	var	rep	rep	t/ha	ST	proteins	N	P_2O_5	P	K_2O	K	Ca	Mg	Fe	Zn	Mn	Cu	Brix
	oznaka za SAS	var	rep1	rep2	prinos_04	ST_04	sirprot_04	N_04	P2O5_04	P_04	K2O_04	K_04	Ca_04	Mg_04	Fe_04	Zn_04	Mn_04	Cu_04	Brix_04
2091	1	**1**	**I**	I	37,5	15,6	9,1	1,46	0,46	0,20	2,69	2,24	0,25	0,25	120	34	51	8,0	13,6
2094	2	**2**	**I**	II	42,7	13,0	9,7	1,55	0,69	0,30	3,38	2,82	0,21	0,20	110	33	40	9,5	10,5
2097	3	**3**	**I**	III	44,1	15,9	10,1	1,61	0,63	0,28	3,46	2,88	0,20	0,25	99	32	44	9,8	12,6
2100	4	**4**	**I**	IV	42,9	16,0	9,5	1,52	0,59	0,26	3,24	2,70	0,24	0,18	108	28	41	11,0	12,9
2103	5	**2**	**II**	IV	46,6	13,2	9,3	1,49	0,77	0,34	3,57	2,98	0,23	0,24	121	30	36	11,0	10
2106	6	**4**	**II**	III	52,7	14,4	11,0	1,76	0,63	0,28	3,02	2,52	0,22	0,24	99	29	36	9,6	11,8
2109	7	**1**	**II**	II	43,1	14,5	9,7	1,55	0,59	0,26	2,76	2,30	0,20	0,21	90	32	40	9,6	12
2112	8	**3**	**II**	I	42,4	13,4	11,4	1,83	0,58	0,25	2,96	2,47	0,19	0,22	108	37	41	9,8	11,6
2115	9	**2**	**III**	I	41,1	13,5	8,9	1,43	0,306	0,13	2,78	2,32	0,16	0,20	99	28	40	8,3	10,5
2118	10	**4**	**III**	II	49,8	13,6	9,9	1,58	0,339	0,15	2,91	2,43	0,19	0,19	99	28	40	8,2	11,9
2121	11	**1**	**III**	III	47,3	13,9	8,8	1,41	0,296	0,13	2,74	2,28	0,34	0,28	116	28	43	8,8	12,3
2124	12	**3**	**III**	IV	46,8	15,0	7,4	1,18	0,314	0,14	2,48	2,07	0,16	0,20	92	28	43	6,9	13,2
2127	13	**1**	**IV**	IV	36,1	15,0	8,3	1,32	0,255	0,11	2,56	2,13	0,21	0,20	108	26	51	9,6	13,4
2130	14	**2**	**IV**	III	40,0	15,9	8,6	1,37	0,283	0,12	2,70	2,25	0,19	0,23	80	26	52	9,1	13,8
2133	15	**3**	**IV**	II	43,2	15,6	8,4	1,34	0,278	0,12	2,57	2,14	0,18	0,18	93	28	49	8,9	13,2
2136	16	**4**	**IV**	I	38,1	18,3	8,4	1,34	0,244	0,11	2,29	1,91	0,21	0,19	90	28	49	8,8	14,7

		yield	%	crude	% in dry matter							mg/kg in dry matter				%
**Average by variants**		t/ha	ST	proteins	N	P_2O_5	P	K_2O	K	Ca	Mg	Fe	Zn	Mn	Cu	Brix
**varijant 1**	unfertilized control	41,0	14,7	9,0	1,4	0,4	0,17	2,7	2,2	0,25	0,23	109	30	46	9,0	12,8
**varijant 2**	stable manure, 50 t/ha	42,6	13,9	9,1	1,5	0,5	0,22	3,1	2,6	0,20	0,22	103	29	42	9,5	11,2
**varijant 3**	500 kg NPK/ha*	44,1	15,0	9,3	1,5	0,5	0,20	2,9	2,4	0,18	0,21	98	31	44	8,9	12,7
**varijant 4**	1000 kg NPK/ha*	45,9	15,6	9,7	1,6	0,5	0,20	2,9	2,4	0,21	0,20	99	28	42	9,4	12,8

formula: broj/(100/ST) formula: broj/(100/ST)

MINERALS IN BEETROOT

2004

ID	sample				yield	%	crude	% in fresh matter							mg/kg in fresh matter				%
	label	var	rep	rep	t/ha	ST	proteins	N	P_2O_5	P	K_2O	K	Ca	Mg	Fe	Zn	Mn	Cu	Brix
	oznaka za SAS	var	rep1	rep2	prinos_04	ST_04	sirprot_04	N_04	P2O5_04	P_04	K2O_04	K_04	Ca_04	Mg_04	Fe_04	Zn_04	Mn_04	Cu_04	Brix_04
1398	1	**1**	**I**	I	37,5	15,6	1,4	0,23	0,07	0,03	0,42	0,35	0,04	0,04	18,66	5,29	7,93	1,24	13,6
1399	2	**2**	**I**	II	42,7	13,0	1,3	0,20	0,09	0,04	0,44	0,37	0,03	0,03	14,29	4,29	5,20	1,23	10,5
1400	3	**3**	**I**	III	44,1	15,9	1,6	0,26	0,10	0,04	0,55	0,46	0,03	0,04	15,75	5,09	7,00	1,56	12,6
1401	4	**4**	**I**	IV	42,9	16,0	1,5	0,24	0,09	0,04	0,52	0,43	0,04	0,03	17,23	4,47	6,54	1,75	12,9
1402	5	**2**	**II**	IV	46,6	13,2	1,2	0,20	0,10	0,04	0,47	0,39	0,03	0,03	15,97	3,96	4,75	1,45	10
1403	6	**4**	**II**	III	52,7	14,4	1,6	0,25	0,09	0,04	0,44	0,36	0,03	0,04	14,29	4,18	5,19	1,39	11,8
1404	7	**1**	**II**	II	43,1	14,5	1,4	0,22	0,09	0,04	0,40	0,33	0,03	0,03	13,04	4,64	5,80	1,39	12
1405	8	**3**	**II**	I	42,4	13,4	1,5	0,25	0,08	0,03	0,40	0,33	0,03	0,03	14,50	4,97	5,51	1,32	11,6
1406	9	**2**	**III**	I	41,1	13,5	1,2	0,19	0,04	0,02	0,37	0,31	0,02	0,03	13,33	3,77	5,38	1,12	10,5
1407	10	**4**	**III**	II	49,8	13,6	1,3	0,21	0,05	0,02	0,40	0,33	0,03	0,03	13,44	3,80	5,43	1,11	11,9
1408	11	**1**	**III**	III	47,3	13,9	1,2	0,20	0,04	0,02	0,38	0,32	0,05	0,04	16,17	3,90	5,99	1,23	12,3
1409	12	**3**	**III**	IV	46,8	15,0	1,1	0,18	0,05	0,02	0,37	0,31	0,02	0,03	13,79	4,20	6,45	1,03	13,2
1410	13	**1**	**IV**	IV	36,1	15,0	1,2	0,20	0,04	0,02	0,38	0,32	0,03	0,03	16,20	3,90	7,65	1,44	13,4
1411	14	**2**	**IV**	III	40,0	15,9	1,4	0,22	0,04	0,02	0,43	0,36	0,03	0,04	12,69	4,12	8,25	1,44	13,8
1412	15	**3**	**IV**	II	43,2	15,6	1,3	0,21	0,04	0,02	0,40	0,33	0,03	0,03	14,54	4,38	7,66	1,39	13,2
1413	16	**4**	**IV**	I	38,1	18,3	1,5	0,24	0,04	0,02	0,42	0,35	0,04	0,03	16,44	5,12	8,95	1,61	14,7

		yield	%	crude	% in fresh matter							mg/kg in fresh matter				%
**Average by variants**		t/ha	ST	proteins	N	P_2O_5	P	K_2O	K	Ca	Mg	Fe	Zn	Mn	Cu	Brix
**varijant 1**	unfertilized control	41,0	14,7	1,3	0,2	0,06	0,03	0,4	0,3	0,0	0,0	16	4	7	1,3	12,8
**varijant 2**	stable manure, 50 t/ha	42,6	13,9	1,3	0,2	0,07	0,03	0,4	0,4	0,0	0,0	14	4	6	1,3	11,2
**varijant 3**	500 kg NPK/ha*	44,1	15,0	1,4	0,2	0,07	0,03	0,4	0,4	0,0	0,0	15	5	7	1,3	12,7
**varijant 4**	1000 kg NPK/ha*	45,9	15,6	1,5	0,2	0,07	0,03	0,4	0,4	0,0	0,0	15	4	7	1,5	12,8

formula: broj*1000 formula: broj/10

MINERALS IN BEETROOT

2004

ID	sample				yield	%	crude	mg/100 g in fresh matter							mg/100 g in fresh matter				%
	label	var	rep	rep	t/ha	ST	proteins	N	P_2O_5	P	K_2O	K	Ca	Mg	Fe	Zn	Mn	Cu	Brix
	oznaka za SAS	var	rep1	rep2	prinos_04	ST_04	sirprot_04	N_04	P2O5_04	P_04	K2O_04	K_04	Ca_04	Mg_04	Fe_04	Zn_04	Mn_04	Cu_04	Brix_04
1398	1	**1**	**I**	I	37,5	15,6	1418,9	227,0	71,5	31,2	418,3	348,6	38,4	38,1	1,87	0,53	0,79	0,12	13,6
1399	2	**2**	**I**	II	42,7	13,0	1258,4	201,3	89,6	39,1	439,1	365,9	27,5	25,7	1,43	0,43	0,52	0,12	10,5
1400	3	**3**	**I**	III	44,1	15,9	1600,9	256,2	100,2	43,8	550,5	458,7	31,2	39,6	1,58	0,51	0,70	0,16	12,6
1401	4	**4**	**I**	IV	42,9	16,0	1515,3	242,4	94,1	41,1	516,8	430,7	37,6	27,9	1,72	0,45	0,65	0,18	12,9
1402	5	**2**	**II**	IV	46,6	13,2	1229,3	196,7	101,6	44,4	471,2	392,7	30,4	31,3	1,60	0,40	0,48	0,15	10
1403	6	**4**	**II**	III	52,7	14,4	1587,3	254,0	90,9	39,7	435,8	363,2	31,6	35,2	1,43	0,42	0,52	0,14	11,8
1404	7	**1**	**II**	II	43,1	14,5	1403,7	224,6	85,5	37,3	399,9	333,3	28,8	30,4	1,30	0,46	0,58	0,14	12
1405	8	**3**	**II**	I	42,4	13,4	1536,1	245,8	77,9	34,0	397,5	331,3	26,1	29,3	1,45	0,50	0,55	0,13	11,6
1406	9	**2**	**III**	I	41,1	13,5	1203,0	192,5	41,2	18,0	374,2	311,8	21,5	26,9	1,33	0,38	0,54	0,11	10,5
1407	10	**4**	**III**	II	49,8	13,6	1341,0	214,6	46,0	20,1	395,2	329,3	25,3	25,7	1,34	0,38	0,54	0,11	11,9
1408	11	**1**	**III**	III	47,3	13,9	1228,5	196,6	41,3	18,0	382,0	318,3	46,8	39,0	1,62	0,39	0,60	0,12	12,3
1409	12	**3**	**III**	IV	46,8	15,0	1105,5	176,9	47,1	20,6	371,8	309,8	24,0	30,0	1,38	0,42	0,64	0,10	13,2
1410	13	**1**	**IV**	IV	36,1	15,0	1237,5	198,0	38,3	16,7	384,0	320,0	31,5	30,0	1,62	0,39	0,77	0,14	13,4
1411	14	**2**	**IV**	III	40,0	15,9	1358,0	217,3	44,9	19,6	428,2	356,9	30,6	35,7	1,27	0,41	0,82	0,14	13,8
1412	15	**3**	**IV**	II	43,2	15,6	1309,0	209,4	43,5	19,0	401,7	334,7	28,1	28,1	1,45	0,44	0,77	0,14	13,2
1413	16	**4**	**IV**	I	38,1	18,3	1530,1	244,8	44,6	19,5	418,4	348,7	38,4	34,5	1,64	0,51	0,90	0,16	14,7

		yield	%	crude	mg/100 g in fresh matter							mg/100 g in fresh matter				%
**Average by variants**		t/ha	ST	proteins	N	P_2O_5	P	K_2O	K	Ca	Mg	Fe	Zn	Mn	Cu	Brix
**varijant 1**	unfertilized control	41,0	14,7	1322	212	59	26	396	330	36	34	1,60	0,44	0,68	0,13	12,8
**varijant 2**	stable manure, 50 t/ha	42,6	13,9	1262	202	69	30	428	357	28	30	1,41	0,40	0,59	0,13	11,2
**varijant 3**	500 kg NPK/ha*	44,1	15,0	1388	222	67	29	430	359	27	32	1,46	0,47	0,67	0,13	12,7
**varijant 4**	1000 kg NPK/ha*	45,9	15,6	1493	239	69	30	442	368	33	31	1,53	0,44	0,65	0,15	12,8

MINERALS IN BEETROOT

2005

ID	sample				yield	%	crude	% in dry matter							mg/kg in dry matter				%
	label	var	rep	rep	t/ha	ST	proteins	N	P_2O_5	P	K_2O	K	Ca	Mg	Fe	Zn	Mn	Cu	Brix
	oznaka za SAS	var	rep1	rep2	prinos_05	ST_05	sirprot_05	N_05	P2O5_05	P_05	K2O_05	K_05	Ca_05	Mg_05	Fe_05	Zn_05	Mn_05	Cu_05	Brix_05
3250	1	**1**	**I**	I	8,0	7,22	19,7	3,15	0,893	0,39	5,07	4,23	0,296	0,25	96,0	45,0	66,0	5,4	11,6
3251	2	**2**	**I**	II	15,0	6,31	21,0	3,36	0,829	0,36	6,43	5,36	0,254	0,27	99,0	43,0	55,0	6,0	10,0
3252	3	**3**	**I**	III	22,2	6,59	18,7	2,99	0,89	0,39	6,24	5,20	0,24	0,23	96,0	42,0	60,0	4,8	10,4
3253	4	**4**	**I**	IV	9,5	6,33	20,6	3,3	0,89	0,39	6,28	5,23	0,283	0,27	105,0	40,0	62,0	5,4	8,9
3254	5	**2**	**II**	IV	14,9	6,28	20,0	3,2	1,074	0,47	6,24	5,20	0,27	0,24	109,0	41,0	56,0	5,6	9,9
3255	6	**4**	**II**	III	26,4	6,10	21,4	3,42	1,07	0,47	6,44	5,37	0,27	0,25	99,0	38,0	59,0	5,0	8,4
3256	7	**1**	**II**	II	22,8	6,31	18,5	2,96	0,81	0,35	5,8	4,83	0,25	0,22	92,0	40,0	55,0	5,6	10,3
3257	8	**3**	**II**	I	20,3	6,01	18,7	2,99	0,848	0,37	5,55	4,63	0,23	0,23	98,0	44,0	56,0	5,2	10,2
3258	9	**2**	**III**	I	32,7	5,88	18,9	3,02	1,09	0,48	5,01	4,18	0,22	0,26	95,0	39,0	56,0	4,6	9,6
3259	10	**4**	**III**	II	38,1	7,28	21,0	3,36	0,729	0,32	5,54	4,62	0,231	0,26	90,0	39,0	59,0	5,6	10,7
3260	11	**1**	**III**	III	28,6	7,91	21,4	3,42	1,00	0,44	4,95	4,13	0,289	0,24	101,0	40,0	60,0	5,6	10,4
3261	12	**3**	**III**	IV	15,0	8,3	18,4	2,94	0,63	0,28	5,07	4,23	0,24	0,23	90,0	39,0	55,0	5,1	11,2
3262	13	**1**	**IV**	IV	10,9	7,78	17,9	2,87	0,66	0,29	5,54	4,62	0,256	0,23	98,0	39,0	66,0	5,4	11,2
3263	14	**2**	**IV**	III	39,6	6,66	19,4	3,1	0,67	0,29	5,29	4,41	0,251	0,23	84,0	38,0	62,0	5,4	9,3
3264	15	**3**	**IV**	II	38,0	7,23	19,6	3,14	0,78	0,34	5,07	4,23	0,292	0,26	89,0	39,0	59,0	5,1	10,6
3265	16	**4**	**IV**	I	29,3	9,45	20,4	3,27	0,73	0,32	4,66	3,88	0,259	0,2	92,0	38,0	60,0	5,0	10,8

		yield	%	crude	% in dry matter							mg/kg in dry matter				%
**Average by variants**		t/ha	ST	proteins	N	P_2O_5	P	K_2O	K	Ca	Mg	Fe	Zn	Mn	Cu	Brix
**varijant 1**	unfertilized control	17,6	7,3	19,4	3,1	0,8	0,4	5,3	4,5	0,3	0,2	96,8	41,0	61,8	5,5	10,9
**varijant 2**	stable manure, 50 t/ha	25,6	6,3	19,8	3,2	0,9	0,4	5,7	4,8	0,2	0,3	96,8	40,3	57,3	5,4	9,7
**varijant 3**	500 kg NPK/ha*	23,9	7,0	18,8	3,0	0,8	0,3	5,5	4,6	0,3	0,2	93,3	41,0	57,5	5,0	10,6
**varijant 4**	1000 kg NPK/ha*	25,8	7,3	20,9	3,3	0,9	0,4	5,7	4,8	0,3	0,2	96,5	38,8	60,0	5,3	9,7

formula: broj/(100/ST) formula: broj/(100/ST)

MINERALS IN BEETROOT

2005

ID	sample				yield	%	crude	% in fresh matter							mg/kg in fresh matter				%
	label	var	rep	rep	t/ha	ST	proteins	N	P_2O_5	P	K_2O	K	Ca	Mg	Fe	Zn	Mn	Cu	Brix
	oznaka za SAS	var	rep1	rep2	prinos_05	ST_05	sirprot_05	N_05	P2O5_05	P_05	K2O_05	K_05	Ca_05	Mg_05	Fe_05	Zn_05	Mn_05	Cu_05	Brix_05
1398	1	**1**	**I**	I	8,0	7,22	1,4	0,23	0,06	0,03	0,37	0,31	0,02	0,02	6,93	3,25	4,77	0,39	11,6
1399	2	**2**	**I**	II	15,0	6,31	1,3	0,21	0,05	0,02	0,41	0,34	0,02	0,02	6,25	2,71	3,47	0,38	10,0
1400	3	**3**	**I**	III	22,2	6,59	1,2	0,20	0,06	0,03	0,41	0,34	0,02	0,02	6,33	2,77	3,95	0,32	10,4
1401	4	**4**	**I**	IV	9,5	6,33	1,3	0,21	0,06	0,02	0,40	0,33	0,02	0,02	6,65	2,53	3,92	0,34	8,9
1402	5	**2**	**II**	IV	14,9	6,28	1,3	0,20	0,07	0,03	0,39	0,33	0,02	0,02	6,85	2,57	3,52	0,35	9,9
1403	6	**4**	**II**	III	26,4	6,10	1,3	0,21	0,07	0,03	0,39	0,33	0,02	0,02	6,04	2,32	3,60	0,31	8,4
1404	7	**1**	**II**	II	22,8	6,31	1,2	0,19	0,05	0,02	0,37	0,30	0,02	0,01	5,81	2,52	3,47	0,35	10,3
1405	8	**3**	**II**	I	20,3	6,01	1,1	0,18	0,05	0,02	0,33	0,28	0,01	0,01	5,89	2,64	3,37	0,31	10,2
1406	9	**2**	**III**	I	32,7	5,88	1,1	0,18	0,06	0,03	0,29	0,25	0,01	0,02	5,59	2,29	3,29	0,27	9,6
1407	10	**4**	**III**	II	38,1	7,28	1,5	0,24	0,05	0,02	0,40	0,34	0,02	0,02	6,55	2,84	4,30	0,41	10,7
1408	11	**1**	**III**	III	28,6	7,91	1,7	0,27	0,08	0,03	0,39	0,33	0,02	0,02	7,99	3,16	4,75	0,44	10,4
1409	12	**3**	**III**	IV	15,0	8,3	1,5	0,24	0,05	0,02	0,42	0,35	0,02	0,02	7,47	3,24	4,57	0,42	11,2
1410	13	**1**	**IV**	IV	10,9	7,78	1,4	0,22	0,05	0,02	0,43	0,36	0,02	0,02	7,62	3,03	5,13	0,42	11,2
1411	14	**2**	**IV**	III	39,6	6,66	1,3	0,21	0,04	0,02	0,35	0,29	0,02	0,02	5,59	2,53	4,13	0,36	9,3
1412	15	**3**	**IV**	II	38,0	7,23	1,4	0,23	0,06	0,02	0,37	0,31	0,02	0,02	6,43	2,82	4,27	0,37	10,6
1413	16	**4**	**IV**	I	29,3	9,45	1,9	0,31	0,07	0,03	0,44	0,37	0,02	0,02	8,69	3,59	5,67	0,47	10,8

Average by variants		yield	%	crude	% in fresh matter							mg/kg in fresh matter				%
		t/ha	ST	proteins	N	P_2O_5	P	K_2O	K	Ca	Mg	Fe	Zn	Mn	Cu	Brix
**varijant 1**	unfertilized control	17,6	7,3	1,4	0,2	0,06	0,03	0,4	0,3	0,020	0,017	7	3	5	0,4	10,9
**varijant 2**	stable manure, 50 t/ha	25,6	6,3	1,2	0,2	0,06	0,02	0,4	0,3	0,016	0,016	6	3	4	0,3	9,7
**varijant 3**	500 kg NPK/ha*	23,9	7,0	1,3	0,2	0,05	0,02	0,4	0,3	0,018	0,017	7	3	4	0,4	10,6
**varijant 4**	1000 kg NPK/ha*	25,8	7,3	1,5	0,2	0,06	0,03	0,4	0,3	0,019	0,018	7	3	4	0,4	9,7

formula: broj*1000 formula: broj/10

MINERALS IN BEETROOT

2005

ID	sample				yield	%	crude	mg/100 g in fresh matter							mg/100 g in fresh matter				%
	label	var	rep	rep	t/ha	ST	proteins	N	P_2O_5	P	K_2O	K	Ca	Mg	Fe	Zn	Mn	Cu	Brix
	oznaka za SAS	var	rep1	rep2	prinos_05	ST_05	sirprot_05	N_05	P2O5_05	P_05	K2O_05	K_05	Ca_05	Mg_05	Fe_05	Zn_05	Mn_05	Cu_05	Brix_05
1398	1	**1**	**I**	I	8,0	7,22	1421,4	227,4	64,5	28,2	366,1	305,0	21,4	18,1	0,69	0,32	0,48	0,04	11,6
1399	2	**2**	**I**	II	15,0	6,31	1325,1	212,0	52,3	22,8	405,7	338,1	16,0	17,0	0,62	0,27	0,35	0,04	10,0
1400	3	**3**	**I**	III	22,2	6,59	1231,5	197,0	58,7	25,6	411,2	342,7	15,8	15,2	0,63	0,28	0,40	0,03	10,4
1401	4	**4**	**I**	IV	9,5	6,33	1305,6	208,9	56,3	24,6	397,5	331,3	17,9	17,1	0,66	0,25	0,39	0,03	8,9
1402	5	**2**	**II**	IV	14,9	6,28	1256,0	201,0	67,4	29,5	391,9	326,6	17,0	15,1	0,68	0,26	0,35	0,04	9,9
1403	6	**4**	**II**	III	26,4	6,10	1303,9	208,6	65,3	28,5	392,8	327,4	16,5	15,3	0,60	0,23	0,36	0,03	8,4
1404	7	**1**	**II**	II	22,8	6,31	1167,4	186,8	51,1	22,3	366,0	305,0	15,8	13,9	0,58	0,25	0,35	0,04	10,3
1405	8	**3**	**II**	I	20,3	6,01	1123,1	179,7	51,0	22,3	333,6	278,0	13,8	13,8	0,59	0,26	0,34	0,03	10,2
1406	9	**2**	**III**	I	32,7	5,88	1109,9	177,6	64,1	28,0	294,6	245,5	12,9	15,3	0,56	0,23	0,33	0,03	9,6
1407	10	**4**	**III**	II	38,1	7,28	1528,8	244,6	53,1	23,2	403,3	336,1	16,8	18,9	0,66	0,28	0,43	0,04	10,7
1408	11	**1**	**III**	III	28,6	7,91	1690,8	270,5	79,1	34,5	391,5	326,3	22,9	19,0	0,80	0,32	0,47	0,04	10,4
1409	12	**3**	**III**	IV	15,0	8,3	1525,1	244,0	52,3	22,8	420,8	350,7	19,9	19,1	0,75	0,32	0,46	0,04	11,2
1410	13	**1**	**IV**	IV	10,9	7,78	1395,5	223,3	51,3	22,4	431,0	359,2	19,9	17,9	0,76	0,30	0,51	0,04	11,2
1411	14	**2**	**IV**	III	39,6	6,66	1290,4	206,5	44,6	19,5	352,3	293,6	16,7	15,3	0,56	0,25	0,41	0,04	9,3
1412	15	**3**	**IV**	II	38,0	7,23	1418,9	227,0	56,4	24,6	366,6	305,5	21,1	18,8	0,64	0,28	0,43	0,04	10,6
1413	16	**4**	**IV**	I	29,3	9,45	1931,3	309,0	69,0	30,1	440,4	367,0	24,5	18,9	0,87	0,36	0,57	0,05	10,8

			yield	%	crude	mg/100 g in fresh matter							mg/100 g in fresh matter				%
**Average by variants**			t/ha	ST	proteins	N	P_2O_5	P	K_2O	K	Ca	Mg	Fe	Zn	Mn	Cu	Brix
**varijant 1**	unfertilized control		17,6	7,3	1419	227	62	27	389	324	20	17	0,71	0,30	0,45	0,04	10,9
**varijant 2**	stable manure, 50 t/ha		25,6	6,3	1245	199	57	25	361	301	16	16	0,61	0,25	0,36	0,03	9,7
**varijant 3**	500 kg NPK/ha*		23,9	7,0	1325	212	55	24	383	319	18	17	0,65	0,29	0,40	0,04	10,6
**varijant 4**	1000 kg NPK/ha*		25,8	7,3	1517	243	61	27	409	340	19	18	0,70	0,28	0,44	0,04	9,7
